# P-value and effect-size in clinical and experimental studies

**DOI:** 10.1590/1677-5449.210038

**Published:** 2021-07-05

**Authors:** Anna Carolina Miola, Hélio Amante Miot

**Affiliations:** 1 Universidade Estadual Paulista – UNESP, Faculdade de Medicina – FMB, Departamento de Infectologia, Dermatologia, Diagnóstico por Imagem e Radioterapia, Botucatu, SP, Brasil.

The complex nature of biological systems causes a certain degree of sample variation in many experiments. Moreover, most biomedical interventions promote moderate effects that do not have an obvious dose-response slope. As a result, when statistics are used to determine the difference between samples, the combination of large measurement variations and modest differences between groups compromises their analytical power (type II error). This means it is imperative to interpret *p*-values (statistical significance) and effect sizes with great care when making inferences from the results of studies that make comparisons between groups, although these concepts are also applicable to analyses of correlation, agreement, survival, and diagnostic tests, among others.^1^^-^5

According to frequentist statistics, two or more samples may be drawn from the same population, but nevertheless show a certain variability in some of their characteristics. The greater the similarity between the samples, the greater the likelihood that they will be of the same nature; while the flip side is that samples that are very different will be less likely to have been chosen at random, from within the same population. Statisticians have developed a series of mathematical models that estimate the probability that samples belong to the same population and the differences observed between them in an experiment have occurred by chance. As a general rule, the *p*-value of a statistical test reflects the theoretical probability that values more extreme than those observed are the result of chance, as long as the groups tested are truly equal (H_0_ is true).^6^^,^^7^

It is the researchers’ responsibility to define a cutoff point beyond which they can consider that the *p*-value denotes a low enough probability that the groups can be assumed to be different. The choice of this significance level (level α) and the decision on the direction of analysis (one-tailed or two-tailed), should be based on theoretical principles and should be defined before the analysis. This is of fundamental importance, because every cutoff point chosen has the potential to sacrifice conclusions derived from results very close to this limit. For example, if the cutoff point chosen is p < 0.05, p = 0.04, it is overvalued in detriment to p = 0.06.^8^

**Figure 1:**
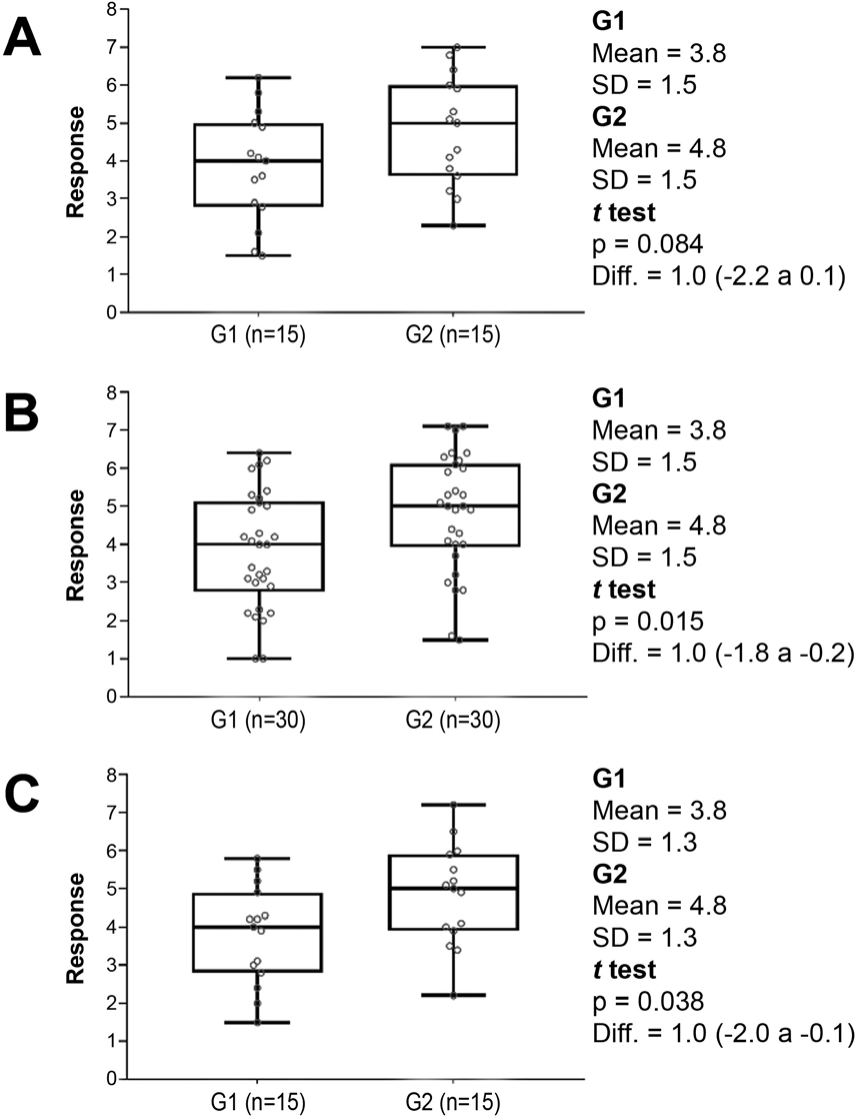
Hypothetical examples of (bidirectional) comparisons between two treatment groups (G1 and G2), all with the same means and medians. (A) Sample with 15 participants per group (p = 0.08); (B) Sample with 30 participants per group and the same standard deviation as in example A (p = 0.02); (C) Sample with 15 participants per group and a smaller standard deviation than example A (p = 0.04).

In tests comparing groups, the *p*-value is influenced by the difference between the means (or proportions), but also by the variance of the data and by the dimensions of the sample. Figure 1 illustrates three different situations, in which samples with variation in standard deviations and sample size are compared. Samples with the same mean and standard deviation have different *p*-values, depending on the sample sizes (Figure 1 A *vs*. B). In turn, samples with the same mean and sample size have different *p*-values if they differ only in terms of their standard deviation (Figure 1 A *vs*. C).

By convention, researchers adopt significance levels in the region of 5% (p ≤ 0.05) for analysis of small samples (n < 50) and, by so doing, accept the risk that the result observed occurs by chance at least once in every 20 times the experiment is run.^9^ Adoption of more stringent significance levels (for example, p < 0.01) increases the reproducibility of studies, but penalizes them with larger type II errors. However, since the sample size and the number of variables involved in the analysis (number of comparisons) influence the *p*-value, this should be carefully weighed up when choosing the significance level. Use of very large samples (n > 1,000) makes finding low *p*-values by chance more likely, so it is recommended that more stringent significance levels be used, such as p ≤ 0.001. Modern genetic experiments simultaneously compare thousands of variables, making detection of small *p*-values by chance more likely, so it is recommended that significance levels of the order of p < 5x10^-8^ should be adopted.^10^^,^^11^

The *p*-values produced by a statistical test should be reported as their exact values, with a number of decimal places compatible with the magnitude that is being evaluated. For example, p = 0.032 should be reported, rather than p < 0.05 or p = 0.032016.^12^^,^^13^ Increasing the number of decimal places is not proof that the results are more important or reliable. Moreover, marginal *p*-values, that are borderline to the significance level (for example, p = 0.067), should not be interpreted as a “trend” to rejection of the null hypothesis, since expanding the sample does not guarantee that the difference between groups will be maintained.^14^

It is, therefore, important that the *p*-value should not be used as a measure of the validity of a result or of the strength of an association.^15^ Neither should *p*-values larger than the significance level (for example, p > 0.1) be interpreted as showing that the samples are identical.^7^ One additional measure for understanding the relationship between the groups sampled is provided by estimators of effect size.^16^

Assuming that the samples are adequately representative of a population (randomized collection), their statistics can be used to estimate parameters of that population, enabling inferences to be made about the behavior of the variables studied. Effect size is an indicator that quantifies the difference between samples, and an estimation of its 95% confidence interval (95%CI) provides a measure of the uncertainty of the behavior of that parameter in the population from which the sample was drawn, providing more valuable information about the true behavior of the phenomenon studied than the *p*-value offers.^17^^,^^18^

Table 1 lists the most important indicators of effect size used in epidemiological studies, which should be presented together with the *p*-value in the results of statistical tests, although the independent meaning of each of them is beyond the scope of this text.^19^ There are other estimators of effect size, which are more often used in experimental studies and which are less intuitive to interpret. These include Cohen’s “d” coefficient “, R^2^, and omega and “eta” squared (ω^2^ and η^2^), which may require help from an experienced statistician.^18^^,^^20^

**Table 1:** Principal measures of effect according to the type of epidemiological study.

**Type of study**	**Effect size**
Diagnostic	Sensitivity, specificity, positive (or negative) predictive value, likelihood ratio, area under the ROC curve
Ecological	Correlation coefficients (r or rho)
Case-control	Odds ratio, prevalence ratio
Survival	Hazard ratio
Clinical trial/cohort study	Relative risk, attributable risk, reduction in relative risk, absolute risk reduction, number needed to treat (or to harm), absolute difference between groups (percentages or means).

ROC = receiver operating characteristic.

Every statistical test should be presented (and interpreted) according to its *p*-value, an effect size, and its 95%CI.^12^^,^^13^^,^^21^^,^^22^ An experiment that results in a large effect size and a *p*-value = 0.06 is undoubtedly more relevant than a result with a small effect size but p < 0.01.^23^^-^25

For example, a recent study that assessed the effectiveness of compression stockings for improving occupational edema found a result with p < 0.0001.^26^ However, the non-availability of reduction values as an effect size (for example, reduction in the diameter of the ankle in the evening, or VEINES scores) makes it difficult to interpret the data and their inferences with a view to clinical use.

Furthermore, particularly when dealing with larger samples, detection of low *p*-values may not indicate a clinically sensitive effect that leads to changes to medical paradigms. In an important systematic review conducted by Martinez-Zapata et al.^27^ on the subject of phlebotonics for venous insufficiency, it was suggested that phlebotonics are superior, on the basis of their statistical significance (p < 0.05), but the effect size observed was the result of a mean reduction of just 4.27 mm (95%CI 2.93–5.61 mm) in ankle circumference in 2,010 participants (15 studies), which, although true, does not indicate an evident benefit for patients with edema of the lower limbs.

Occasionally, there may be a discrete divergence between the amplitude of the effect size and the *p*-value. For example, a relative risk of 0.70 (95%CI 0.36–1.01) and a *p*-value = 0.045. However, this should not be considered an error, since the estimates originate from different calculations and tend to converge as sample sizes increase.

There is a recent academic movement in favor of total abolition of *p*-values and of the term “statistically significant” from scientific publications, giving preference to exclusively reporting the effect size of a test, because it is more informative and allows generalization of results.^28^ Undoubtedly, studies that base their conclusions entirely on the *p*-value are more susceptible to non-reproducibility, in addition to encouraging researchers to pursue statistical significance in detriment to the relevance of the result (“p-hacking”).^23^^,^^28^^-^31 However, this is still an incipient movement among researchers, since a campaign for correct interpretation of *p*-values analyzed in conjunction with effect sizes is a more correct option than abolishing p-values.^32^^,^^33^

Finally, comparisons between groups can be assessed either unidirectionally or bidirectionally (one-tailed or two-tailed). A test is usually called a difference study if we are assessing the behavior of a variable that can be larger or smaller between samples. However, many assessments are by their nature unidirectional, such as a comparison of the number of cases of a disease between people who have been vaccinated and those who have not; or a test of non-inferiority comparing two treatments.^34^ In these examples, the possibility that the result could be considered bidirectionally is not part of the research hypothesis. However, use of one-tailed analyses is not consensus among epidemiologists, because, although they have greater statistical power and need smaller sample sizes, they increase the chance of type I error.^35^^-^37 These analyses require supervision by an experienced statistician to calculate the one-tailed *p*-value and 95%CI.

While the size of the *p*-value can inform a reader whether there is a significant effect, it does not reveal the extent of the impact of this effect on the variables studied.^38^ Researchers must therefore be cautious about the results of statistical tests, in the sense that the p-value should be interpreted in conjunction with the effect size, in particular as estimated by the 95% confidence interval, since the pragmatic significance of an experiment is an information that is independent of its statistical significance.
