# Supplementary material for: P-value and effect-size in clinical and experimental studies
Source: J Vasc Bras. 2021 Jul 5;20:e20210038. doi: 10.1590/1677-5449.210038 (PMC8256876; doi:10.1590/1677-5449.210038)
Supplement: Translation

# Translation: P-valor e dimensão do efeito em estudos clínicos e experimentais

**Authors:** Anna Carolina Miola, Hélio Amante Miot

**Affiliations:** 1 Universidade Estadual Paulista – UNESP, Faculdade de Medicina – FMB, Departamento de Infectologia, Dermatologia, Diagnóstico por Imagem e Radioterapia, Botucatu, SP, Brasil.

A natureza complexa dos sistemas biológicos faz com que muitos experimentos apresentem certa variabilidade amostral. Ainda, grande parte das intervenções biomédicas promove efeitos moderados e sem um evidente gradiente dose-resposta. Contudo, ao passo que se emprega a estatística para concluir quanto à diferença entre amostras, a maior variabilidade das medidas e a modesta diferença entre grupos comprometem o poder analítico (erro tipo II). Esse detalhe exige uma cuidadosa interpretação do p-valor (significância estatística) e da dimensão do efeito na inferência resultante de estudos de comparação entre grupos, apesar desses conceitos se aplicarem também a análises de correlação, concordância, sobrevivência, testes diagnósticos, entre outros^1^^-^5.

Segundo a estatística frequentista, duas ou mais amostras podem ser originárias de uma mesma população, porém, apresentam certa variabilidade em algumas características. Quanto mais similares forem as amostras, maior a chance de terem a mesma natureza; por outro lado, amostras que se apresentam de forma muito diferente têm menor chance de terem sido selecionadas ao acaso, dentro da mesma população. Os estatísticos desenvolveram uma série de modelos matemáticos que estimam a probabilidade de que amostras pertençam a uma mesma população e que suas diferenças constatadas no experimento tenham ocorrido ao acaso. De forma geral, o p-valor de um teste estatístico retorna à probabilidade teórica de que valores mais extremos do que os encontrados sejam frutos do acaso, desde que os grupos testados sejam realmente iguais (H_0_ verdadeira)^6^^,^^7^.

Cabe ao pesquisador definir o ponto de corte a partir do qual ele considera, para o p-valor, uma probabilidade baixa o suficiente para assumir que os grupos sejam diferentes. A decisão desse nível de significância (nível α), assim como a direção da análise (uni ou bicaudal), devem ser baseadas em princípios teóricos e definidas previamente à análise. Isso é de fundamental importância, porque toda escolha de um ponto de corte pode sacrificar conclusões derivadas de resultados muito próximos a esse limite. Por exemplo, não se deve sobrevalorizar p = 0,04 em detrimento de p = 0,06, quando o ponto de corte escolhido for p < 0,05^8^.

Em testes de comparação entre grupos, o p-valor é influenciado pela diferença entre as médias (ou proporções), mas também pela variância dos dados e pela dimensão da amostra. A Figura 1 mostra três situações diferentes, em que se comparam amostras com variação nos desvios-padrão e tamanho amostral. Amostras com mesma média e desvio padrão apresentam p-valores diferentes, de acordo com o tamanho amostral (Figura 1 A *vs*. B). Já amostras com a mesma média e tamanho amostral apresentam p-valores distintos se diferirem apenas quanto ao desvio padrão (Figura 1 A *vs*. C).

**Figura 1:** Exemplos hipotéticos de comparações (bidirecionais) de dois grupos de tratamento (G1 e G2), todos com mesma média e mediana. (A) Amostra com 15 participantes por grupo (p = 0,08); (B) Amostra com 30 participantes por grupo e mesmo desvio padrão que o Exemplo A (p = 0,02); (C) Amostra com 15 participantes por grupo e menor desvio padrão que o exemplo A (p = 0,04).

Convencionalmente, pesquisadores adotam níveis de significância na faixa de 5% (p ≤ 0,05) para a análise de pequenas amostras (n < 50) e, com isso, assumem o risco de o resultado encontrado ocorrer ao acaso em pelo menos uma vez a cada 20 execuções do experimento^9^. A adoção de níveis de significância mais restritos (por exemplo, p < 0,01) aumenta a reprodutibilidade dos estudos, porém, deve penalizá-los com maiores erros do tipo II. Contudo, como o tamanho amostral e o número de variáveis envolvidas na análise (número de comparações) influenciam o p-valor, isso deve ser cuidadosamente ponderado na decisão do nível de significância. O emprego de amostras vultosas (n > 1.000) favorece o encontro ocasional de p-valores pequenos, sendo recomendado utilizar níveis de significância mais restritos, como p ≤ 0,001. As modernas explorações genéticas comparam, simultaneamente, milhares de variáveis, favorecendo o encontro casual de p-valores diminutos, sendo recomendados níveis de significância da ordem de p < 5x10^-8^.^10^^,^^11^

Os p-valores resultantes de um teste estatístico devem ser apresentados como sua medida exata e com um número de decimais compatível com a grandeza que se propõe avaliar. Por exemplo, deve-se referir p = 0,032 em vez de p < 0,05 ou de p = 0,032016^12^^,^^13^. O acréscimo de decimais não é contraprova de maior importância ou fidedignidade dos resultados. Ainda, p-valores marginais ao nível de significância (por exemplo, p = 0,067) não devem ser interpretados como uma “tendência” para rejeitar a hipótese nula, uma vez que a ampliação da amostra não garante que a diferença entre os grupos seja mantida^14^.

É, pois, importante que o p-valor não seja utilizado como medida de validade de um resultado ou da força de uma associação^15^. Tampouco p-valores maiores que o nível de significância (por exemplo, p > 0,1) devem ser interpretados como identidade entre as amostras^7^. Uma medida adicional para a compreensão da relação entre os grupos amostrados são os estimadores chamados de dimensão do efeito^16^.

Desde que as amostras representem adequadamente uma população (coleta aleatorizada), suas estatísticas podem estimar parâmetros dessa mesma população, permitindo realizar inferências sobre o comportamento das variáveis estudadas. A dimensão do efeito é um indicador que quantifica a diferença entre as amostras, e a estimativa do seu intervalo de confiança de 95% (IC95%) dimensiona a incerteza do comportamento do parâmetro na população de origem, retornando uma informação mais valiosa que o p-valor quanto ao real comportamento do fenômeno estudado^17^^,^^18^.

A Tabela 1 apresenta os principais indicadores de dimensão de efeito utilizados em estudos epidemiológicos e que devem acompanhar o p-valor nos resultados de testes estatísticos, contudo, o significado independente de cada um deles ultrapassa o escopo do texto^19^. Há, ainda, outros estimadores de dimensão de efeito, mais empregados em estudos experimentais, cuja interpretação é menos intuitiva; entre eles, estão o coeficiente “d” de Cohen, R^2^, o ômega e o “eta” quadrado (ω^2^ e η^2^), que podem requerer suporte de um estatístico experiente^18^^,^^20^.

**Tabela 1:** Principais dimensões de efeito de acordo com o tipo do estudo epidemiológico.

**Tipo de estudo**	**Dimensão do efeito**
Diagnóstico	Sensibilidade, especificidade, valor preditivo positivo (ou negativo), razão de verossimilhança, área sob a curva ROC
Ecológico	Coeficientes de correlação (r ou rho)
Caso-controle	Razão de chances, razão de prevalência
Sobrevivência	Razão de risco
Ensaio clínico/coorte	Risco relativo, risco atribuível, redução do risco relativo, redução absoluta do risco, número necessário para o tratamento (ou para dano), diferença absoluta entre os grupos (percentual ou médias).

ROC = característica de operação do receptor.

Todo teste estatístico deve ser apresentado (e interpretado) de acordo com o p-valor, uma dimensão do efeito, e seu IC95%^12^^,^^13^^,^^21^^,^^22^. Um experimento que resulte em grande dimensão de efeito e p-valor = 0,06 é certamente mais relevante que um resultado que exiba pequena dimensão do efeito e p < 0,01^23^^-^25.

Um estudo recente que avaliou a efetividade de meias de compressão na melhora do edema ocupacional resultou em p < 0,0001^26^, todavia, a indisponibilidade dos valores de redução como dimensão do efeito (por exemplo, redução do diâmetro vespertino do tornozelo, ou escore VEINES) dificulta a interpretação dos dados e sua inferência visando a indicação clínica.

Por outro lado, especialmente, em amostragens mais vultosas, o encontro de p-valores reduzidos pode não representar em um efeito clinicamente sensível que leve à mudança de paradigmas médicos. Na importante revisão sistemática de Martinez-Zapata et al.^27^ sobre ventonônicos em insuficiência venosa, foi sugerida a superioridade de drogas venotônicas devido a sua significância estatística (p < 0,05), porém, a dimensão do efeito encontrada resultou em uma redução média de apenas 4,27 mm (IC95% 2,93–5,61 mm) na circunferência do tornozelo de 2.010 participantes (15 estudos), o que, apesar de verdadeiro, não representa um benefício evidente para o paciente com edema dos membros inferiores.

Excepcionalmente, pode haver uma discreta divergência entre a amplitude da dimensão de efeito e o p-valor, por exemplo, como um resultado de risco relativo 0,70 (IC95% 0,36–1,01) e p-valor = 0,045, porém, isso não deve ser considerado um erro, já que são estimativas oriundas de cálculos diferentes e que tendem a convergir com o aumento amostral.

Há um recente movimento acadêmico para a completa abolição do p-valor e do termo “estatisticamente significante” nas publicações científicas, em preferência pela representação exclusiva da dimensão de efeito de um teste, por ser mais informativa e permitir a generalização dos resultados^28^. Realmente, estudos que baseiam suas conclusões unicamente no p-valor são mais susceptíveis à não reprodutibilidade, além de estimularem os pesquisadores a perseguirem a significância estatística em detrimento à relevância do resultado (*p-hacking*)^23^^,^^28^^-^31. Contudo, esse ainda é um movimento incipiente entre os pesquisadores, e uma campanha para a interpretação correta do p-valor analisado em combinação com a dimensão do efeito constitui uma alternativa mais acertada que sua abolição^32^^,^^33^.

Finalmente, as comparações entre grupos podem ser avaliadas de forma uni ou bidirecional (uni/bicaudal). Convenciona-se chamar de estudo de diferença quando avaliamos se o comportamento de uma variável pode ser superior ou inferior entre as amostras. Entretanto, muitas avaliações são, por natureza, unidirecionais, como a comparação do número de casos de uma doença entre vacinados e não vacinados; ou em testes de não inferioridade entre duas terapias^34^. Nesses exemplos, não faz parte da hipótese de pesquisa a possibilidade de que o resultado seja contemplado de forma bidirecional. O emprego de análises unicaudais, todavia, não é consensual entre os epidemiologistas, porque, apesar de apresentarem maior poder estatístico e demandarem menor amostragem, aumentam a chance de erro tipo I^35^^-^37. Tais análises exigem supervisão de um estatístico experiente para o cálculo do p-valor e do IC95% unicaudais.

Enquanto a dimensão do p-valor pode informar ao leitor se há algum efeito significativo, o mesmo não revela a extensão do impacto desse efeito nas variáveis estudadas^38^. Portanto, o pesquisador deve estar atento aos resultados dos testes estatísticos, no sentido de que sua interpretação deva contemplar o p-valor em conjunto com a dimensão do efeito, especialmente estimada pelo seu intervalo de confiança de 95%, já que o significado pragmático do experimento é uma informação independente da sua significância estatística.
